# Quantitative Assessment of Road Performance of Recycled Asphalt Mixtures Incorporated with Steel Slag

**DOI:** 10.3390/ma15145005

**Published:** 2022-07-19

**Authors:** Zipeng Wang, Shaopeng Wu, Chao Yang, Jun Xie, Yongli Xiao, Zenggang Zhao, Fusong Wang, Lei Zhang

**Affiliations:** 1 Key Laboratory of Road and Traffic Engineering of the Ministry of Education, Tongji University, Shanghai 201804, China; wangzipeng_sjz@126.com; 2 State Key Laboratory of Silicate Materials for Architectures, Wuhan University of Technology, Wuhan 430070, China; xiejun3970@whut.edu.cn (J.X.); zhaozenggang@whut.edu.cn (Z.Z.); 3 Baoshan Iron & Steel Co., Ltd., Shanghai 201900, China; xiaoyl@baosteel.com; 4 School of Civil Engineering and Mechanics, Huazhong University of Science and Technology, Wuhan 430074, China; wangfs@whut.edu.cn; 5 Department of Civil and Environmental Engineering, Norwegian University of Science and Technology, 7491 Trondheim, Norway; lei.zhang@ntnu.no

**Keywords:** steel slag, RAP, recycled asphalt mixtures, road performance, radar chart evaluation

## Abstract

Circular utilization of reclaimed asphalt pavement (RAP) has received extensive attention for its economic and environmental benefits. The application of recycled asphalt mixtures (RAM) in the upper layer of asphalt pavement faces the issue of inferior anti-slip performance and durability. This study aims to recycle steel slag as virgin aggregates in RAM and quantitatively evaluate the service performance of RAM with steel slag. Steel slag and basalt RAM were firstly fabricated and the five different RAP contents were involved. Then tests of Marshall stability, indirect tensile strength and Cantabro spatter loss were conducted to investigate the moisture susceptibility of RAM. Moreover, their high temperature stability, crack resistance and skid resistance were characterized. Indirect tensile fatigue test combined with Hamburg wheel tracking test were carried out to discuss the durability of RAM. The comprehensive performance of RAM with steel slag were quantitatively assessed based on an improved radar chart evaluation method. The results show that involving steel slag reveals a remarkable enhancement function on water stability, high and low temperature performance, skid resistance and fatigue resistance of RAM. Steel slag RAM with 50% RAP content demonstrates a rutting depth of 7.60 mm and a creep slope of 2.54 × 10^−4^, indicating its superior durability in high temperature and water environment. Compared with the comprehensive evaluation function of 0.5336 for basalt RAM with 30% RAP dosage, steel slag RAM reaches 0.7801, which represents its preferable road performance.

## 1. Introduction

With the dual development of economy and society, China’s highway construction has also entered a stage of rapid development [1]. Asphalt pavement occupies 95% proportion of high-grade roads due to its series of merits [2]. However, asphalt pavement is prone to the diseases of cracks, rutting and water damage, etc. [3]. By the end of 2020, the highway maintenance mileage was 5.14 million km, accounting for 99.0% of the total highway mileage, which implied that China’s highway construction has stepped into the construction and maintenance period [4]. A large amount of reclaimed asphalt pavement (RAP) materials were produced in repair projects, and the recycling of RAP could alleviate resource dependence, energy conservation and emission reduction, which coincided with sustainable development in the transportation industry [5].

The performance of recycled asphalt mixtures was influenced by the material properties and the recycling technology [6,7]. The different sources of RAP had been corroborated to have significant variability in RAM performance [8]. A higher mixing temperature for hot in-place recycling asphalt mixtures revealed superior cracking and moisture resistance [9]. The mixing procedures also showed prominent impact on air voids (AV) and voids in mineral aggregate (VMA) [10]. Chen et al. surveyed the effect of RAP on the performance of recycled asphalt mixtures and found that incorporating RAP improved the resistance to water damage and rutting in contrast to anti-crack performance [11]. Yin et al. endorsed the fact that the moisture stability of recycled asphalt mixtures revealed a descending trend with the addition of RAP. He pointed that hot-mix recycled asphalt mixture (HRAM) exhibited a greater fatigue life than new asphalt mixture (NAM), and its fatigue life increased with the ascending RAP dosage [12]. This is due to generation of a loading buffer interface between aged asphalt and rejuvenator. HRAM had high sensitivity of temperature and strain, resulting in its greater decrement of fatigue life than NAM. These conclusions differed from most of the research results [13,14]. Roja et al. illustrated that the asphalt mixture with 35% RAP content revealed the highest dynamic modulus the least fracture resistance [15]. Zhang et al. found that the recycled asphalt mixture had a lower fatigue life than new asphalt mixture and the gap was enlarged with the ascending RAP content [16]. Some approaches to characterize and classify the RAP were applied to promote its popularization and utilization [17]. Chen et al. investigated the mechanism and performance of mixtures with 100% dosage RAP and bio-rejuvenated additive (BRA) and found that BRA restores the balance of the components of aged bitumen and elevate mechanical performance of asphalt mixtures [18]. Similar studies had also been reported by Zaumanis et al. [19]. Moreover, the controversial moisture susceptibility and fatigue durability of the recycled asphalt mixture were the key elements affecting its implement and application.

Currently, some modifiers are being added to the recycled asphalt and recycled asphalt mixtures to enhance their performance. It was reported that anti-stripping agents was added to recycled asphalt mixtures and demonstrated an outstanding promotion function on rutting resistance and water stability [20]. Zhu et al. explored the fatigue performance of recycled asphalt containing warm mix asphalt (WMA) additive and suggested that the WMA additive could elevate the fatigue potential under stress-loading and strain-loading modes [21]. The fiber was introduced to emulsified asphalt cold recycled mixture for improving its inferior early strength and crack resistance [22]. Kim et al. analyzed the effect of desulfurized gypsum (DG) additive on the mechanical behavior of cold recycled asphalt mixtures (CRAM) [23]. The results stated that DG additive elevated the early strength and stiffness and declined the viscoelasticity of CRAM. Yang et al. considered that cold-recycled mixture with asphalt emulsion (CMAE) with high cement dosage demonstrated larger indirect tensile strength and critical strain energy density [24]. The addition of modifiers brings about high preparation costs and poor storage stability, and it is desirable to improve the crack resistance and durability of recycled asphalt mixtures by involving alternative aggregates with excellent performance.

Steel slag, as the highest amount of solid waste generated and disposed in metallurgical industry, reveals the vantages of anti-slip and abrasion resistance and high adhesion strength [25]. It was concluded that steel slag could be applied as aggregate for road engineering and displayed a remarkable improvement on the performance of asphalt mixture [26]. It was attributed to the fact that steel slag revealed higher polished value and rougher surface texture [27]. Rodríguez-Fernández et al. surveyed the performance parameters of asphalt mixtures with the simultaneous incorporation of RAP and electric arc furnace (EAF) slag [28]. The results proved that recycled asphalt mixtures with EAF slag exhibited a satisfactory resistance to permanent deformation due to excellent polishing resistance and low abrasion coefficient of slag. Pasetto et al. conducted the fatigue analysis of base-binder asphalt mixtures with EAF slag and RAP and found that the stiffness and fatigue resistance of the mixtures was improved due to the addition of EAF slag [29]. Meanwhile, the incorporation of EAF slag also boosted the resilient modulus and dynamic creep property of warm mix asphalt (WMA) mixtures [30]. Yang et al. illustrated that involving steel slag in asphalt mixtures with high RAP content exhibited higher texture depth and BPN than that of basalt [31]. This is closely related to the stronger interlocking structure in steel slag [32].

**Figure 1:**
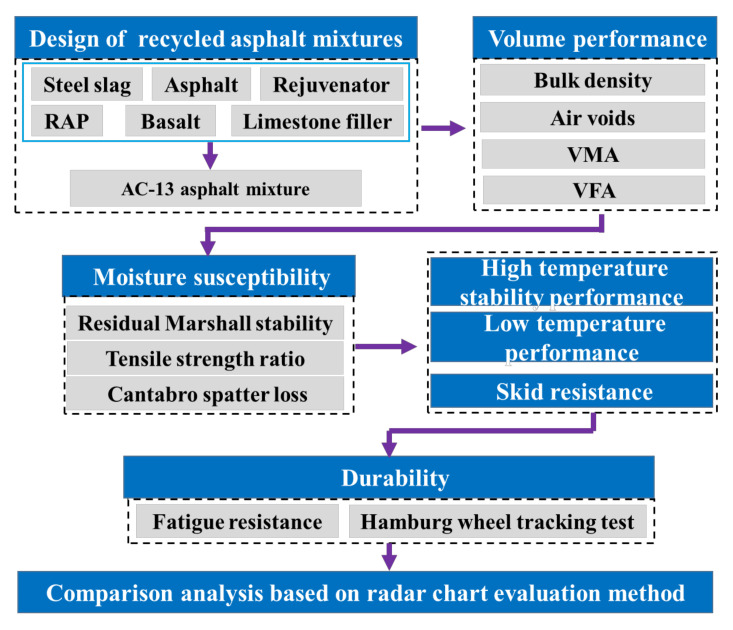
The experimental program of this research.

Overall, incorporating steel slag embodies a commendable performance indexes of recycled asphalt mixtures. Little attention has been paid to the quantitative evaluation of comprehensive performance of recycled asphalt mixtures. The purpose of this research is to explore the service performance of recycled asphalt mixtures incorporated with steel slag, including the volume performance, moisture susceptibility, high temperature stability performance, low temperature performance, skid resistance and durability. Furthermore, an improved radar chart evaluation method was used to quantitatively assess their comprehensive performance. The experimental program is shown in Figure 1. The research results contribute to eliminate the resource dependence and environmental pressure.

## 2. Materials and Methods

### 2.1. Materials

Styrene–butadiene–styrene modified asphalt (named as SBS asphalt for short) was used in this research, which was acted as virgin asphalt. This was mainly because the recycled asphalt mixtures (RAM) in this paper was mainly used in the upper layer of the asphalt pavement. The upper layer usually had strict requirements on the performance of asphalt mixtures, thus SBS asphalt was commonly adopted [33]. SBS asphalt has a penetration of 68 dmm at 25 °C and a ductility of 486 mm at 5 °C and a softening point of 56 °C. Rejuvenator with a density of 0.943 g/cm^3^ and a viscosity of 1.780 Pa·s at 60 °C were included. SBS asphalt and rejuvenator were obtained from Hohhot and Nanjing, China. Steel slag and basalt as virgin aggregates were supplied from Wuhan and Jingshan. Steel slag coupled with SBS modified asphalt were conducted to prepare RAM with excellent performance, so as to create implementation of the maximum additional value of steel slag. The rejuvenator in this study was mainly used to restore the performance index of aged asphalt in RAP [34]. Their properties were displayed in Table 1.

### 2.2. Methods

#### 2.2.1. Preparation of RAM

**Figure 2:**
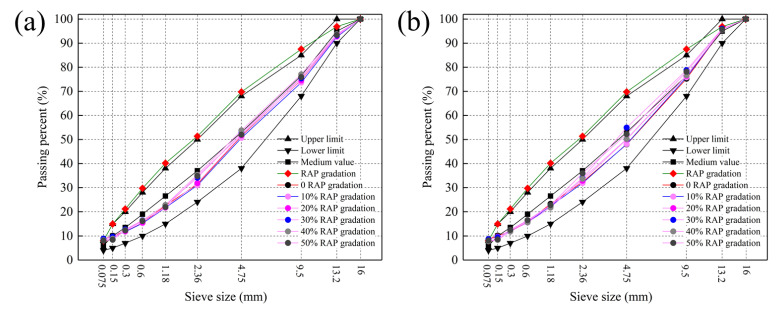
Grading curve of asphalt concrete (AC)-13: (**a**) steel slag; (**b**) basalt.

RAP were acquired from surface pavement on Wu-Huang highway of China. RAM with 10%, 20%, 30%, 40% and 50% RAP and 13.2 mm nominal maximum size were prepared based on Marshall design method. The dosage of the rejuvenator in RAM was 6 wt% (mass ratio of rejuvenator and aged asphalt) [35]. Steel slag and basalt were conducted as virgin coarse aggregates and limestone was conducted as virgin fine aggregate to fabricate two types of RAM. Limestone powder as filler was involved. Figure 2 depicted the grading curve of RAM with steel slag and basalt.

#### 2.2.2. Performance Evaluation of Recycled Asphalt Mixtures

The bulk density, air voids, VMA and VFA were employed to investigate the volume performance of RAM as the evaluation indexes. Tests of Marshall stability, indirect tensile strength and Cantabro spatter loss were applied to assess the moisture susceptibility of RAM. The standard wheel tracking test with a rolling speed of 42 cycles/min, a testing temperature of 60 °C and a load strength of 0.7 MPa were conducted to discuss the high temperature stability performance. Low temperature performance was characterized through a three-points bending test with a temperature of −10 °C and a loading rate of 50 mm/min. Beam specimens with a size of 250 × 30 × 35 mm were involved. Texture depth (TD) and British Pendulum Number (BPN) were used to evaluate the skid resistance.

**Figure 3:**
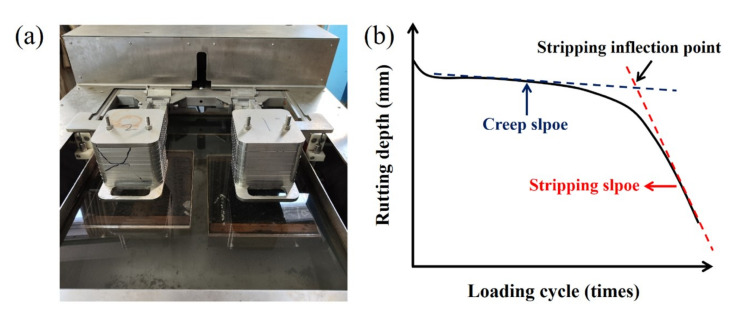
Testing device (**a**) and schematic diagram (**b**) of HWT test.

A fatigue resistance test combined with a Hamburg wheel tracking (HWT) test were carried out to study the durability of RAM. Indirect tensile fatigue test with a testing temperature of 15 °C, the stress level of 0.35, 0.40, 0.45 and 0.50 MPa and a Poisson’s ratio of 0.35 were used. The testing device and schematic diagram of HWT test are described in Figure 3. The creep slope and stripping slope were slope of the tangent line of the creep curve and stripping curve. By calculating the intersection point of the two curves, the stripping inflection point (SIP) could be acquired. The testing temperature and rolling rate were 50 °C and 45 cycles/min [36].

#### 2.2.3. Radar Chart Evaluation Method

The radar chart method is a common graphical method to display multiple variables, which can map a multidimensional space point to two-dimensional space, indicating the feature of qualitative evaluation of each evaluation object. In the traditional radar map evaluation, the area and perimeter of the graph were extracted as feature vectors, while the feature vector area and perimeter revealed the disadvantage of varying with the ranking of indicators. Therefore, an improved radar chart evaluation method with uniqueness feature was conducted to quantitatively assess the comprehensive performance of RAM.

In the improved radar chart evaluation method, the evaluation vector and evaluation function were constructed by extracting feature vectors to comprehensively reflect the level of RAM and the balanced development degree of each index [37]. Firstly, a matrix A = (*a_ij_*)n × k for the evaluation indicators was established. Vector X = {*x*_1_, *x*_2_, *x*_3_…*x*_n_} and Y = {*y*_1_, *y*_2_, *y*_3_…*y*_k_} represent a group of objects and a set of indicators for the objects.

Secondly, the data in matrix A were standardized and non-linear transformed through Equations (1) and (2).

(1) $$b_{ij}=\frac{a_{ij}-E\left(y_{j}\right)}{\sigma \left(y_{j}\right)}$$

(2) $$r_{ij}=\frac{2}{\pi }\arctan\left(b_{ij}\right)+1$$

where *b_ij_* and *r_ij_* represent each indicator after standardization and non-linear transformation, respectively, and *E(y_j_)* and *σ(y_j_)* are the average value and standard deviation indicator *j*.

Thirdly, the characteristic vectors were calculated according to Equations (3) and (4).

(3) $$u_{i}=\left[A_{i},L_{i}\right]$$

(4) $$\left\{\begin{array}{c} A_{i}=\sum_{j=1}^{k}\frac{1}{k}\pi r_{ij}^{2}\\ L_{i}=\sum_{j=1}^{k}\frac{2}{k}\pi r_{ij} \end{array}\right.$$

where *A_i_* and *L_i_* represent the area inside the arcs and sum length of arcs, respectively, and k represents the number of indicators. 

Fourthly, the evaluation vector is defined based on the extracted characteristic vector, as shown in Equation (5).

(5) $$\nu_{i}=\left[\nu_{i1},\nu_{i2}\right]$$

where *ν_i_*_1_ and *ν_i_*_2_ are the relative area and perimeter of evaluation object. Calculation method of *ν_i_*_1_ and *ν_i_*_2_ are displayed in Equation (6).

(6) $$\left\{\begin{array}{c} \nu_{i1}=\frac{A_{i}}{MaxA_{i}}\\ \nu_{i2}=\frac{L_{i}}{2\pi \sqrt{\frac{A_{i}}{\pi }}} \end{array}\right.$$

Finally, the comprehensive evaluation function (f) was deduced through the geometric mean of *ν_i_*_1_ and *ν_i_*_2_, as displayed in Equation (7).

(7) $$f\left(\nu_{i1},\;\nu_{i2}\right)=\sqrt{\nu_{i1}\times \nu_{i2}}$$

The road performance indexes of the RAM were obtained through the performance test. Through the above formula, the evaluation indicators of the two RAM with different RAP content were standardized and normalized, and the comprehensive performance of the two materials was evaluated by the obtained evaluation function.

## 3. Results and Discussion

### 3.1. Volume Performance

The volume performance parameters of RAM are presented in Table 2. When RAP content is lower than 30%, asphalt aggregate ratio of RAM with steel slag no change with the rising RAP content. As the RAP dosage continues to increase, asphalt aggregate ratio rises. The addition of RAP significantly reduces the bulk density of RAM with steel slag, while fluctuates little on the air voids, VMA and VFA. The asphalt-aggregate ratio of RAM with basalt demonstrates the similar change trend as the RAP content increases. There is a certain degree of increase in bulk density of RAM incorporated with basalt as the ascending RAP dosage. This is due to the diminution of virgin fine aggregate content of limestone with lower density. In general, the volume performance of steel slag and basalt RAM with different RAP content meet the specification requirements [38].

### 3.2. Moisture Susceptibility

#### 3.2.1. Residual Marshall Stability

**Figure 4:**
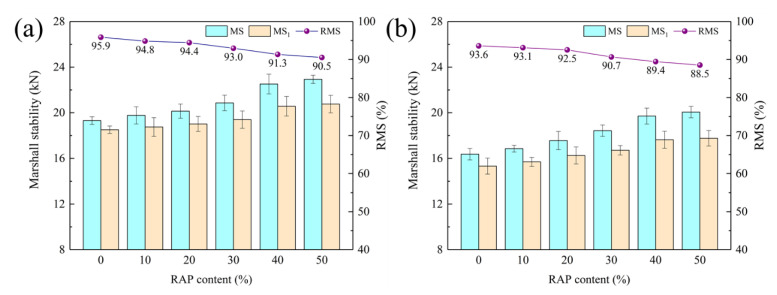
RMS results of RAM: (**a**) steel slag; (**b**) basalt.

Figure 4 illustrates the results of residual Marshall stability (RMS) of RAM incorporated with steel slag and basalt. Compared with the virgin steel slag asphalt mixtures, the Marshall stability (MS) and immersion Marshall stability (MS_1_) of RAM increase with ascending RAP dosage in contrast to RMS. This is attributed to the enhancement of the overall elasticity of RAM with aged asphalt of high modulus. The result is match to the conclusion of Oldham et al. [39]. Meanwhile, aged asphalt reveals inferior adhesion property with aggregates, resulting in the reduction in RMS of RAM as RAP content increases. Steel slag RAM with 50% RAP exhibits an RMS of 90.5% and still remains at a high level. For basalt RAM, its variation rule of MS, MS_1_ and RMS are consistent with that of steel slag RAM. While it demonstrates lower performance indexes under the same RAP content. RAM with steel slag and basalt all satisfy the requirements that the RMS of the modified asphalt mixture in the wet areas is not less than 85%.

#### 3.2.2. Tensile Strength Ratio

**Figure 5:**
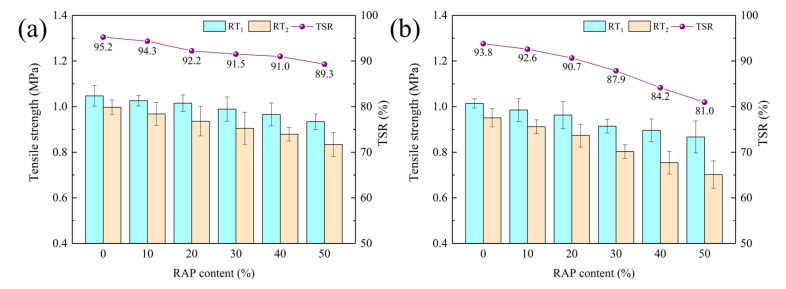
TSR results of RAM: (**a**) steel slag; (**b**) basalt.

Freeze-thaw splitting test can more truly reflect the water damage resistance of asphalt mixtures. Tensile strength ratio (TSR) reveals more stringent requirement than RMS, and TSR of asphalt mixture will not meet the requirements when RMS arrivals design constrain. Figure 5 presents the TSR results of RAM. It is stated that splitting tensile strength and TSR of RAM show a linear decrease with the ascending RAP dosage. This is because that RAP increases the modulus of asphalt in RAM, which diminishes the bonding force between asphalt and aggregate, resulting in the exfoliation of asphalt from the surface layer of the aggregate under water immersion, thus weakening the mechanical properties of the RAM [40]. Steel slag RAM with 50% RAP content exhibits a 6.2% reduction compared to virgin asphalt mixtures and reaches to 89.3%. While for RAM prepared with basalt, the corresponding values are 13.6% and 81.0%. This indicates that incorporating steel slag in RAM can reduce the potential moisture damage risks and elevate the water stability of RAM. This is consistent with the fact that steel slag embodied superior adhesive effect than basalt according to the analysis of molecular simulation [41].

#### 3.2.3. Cantabro Spatter Loss

The Cantabro spatter loss test is commonly applied to assess the adhesion between asphalt and aggregate in open-graded asphalt mixtures. Although a dense gradation was included in this study, the relative high RAP content may reveal a greater impact on the overall bonding of the asphalt mixtures due to inferior adhesion property between aged asphalt and aggregates. Given that AC-13 RAM was adopted as top layer of asphalt pavement, it suffers from surface stripping due to the dual action of rutting and rainwater. Therefore, Cantabro spatter loss can be conducted to characterize resistance to water damage of RAM.

**Figure 6:**
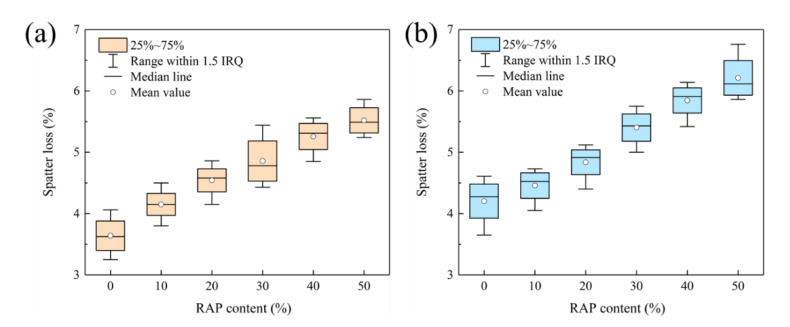
Spatter loss results of RAM: (**a**) steel slag; (**b**) basalt.

Figure 6 depicts the Cantabro spatter loss results of RAM with steel slag and basalt. The spatter loss of steel slag and basalt RAM all boost as the ascending RAP dosage, while steel slag RAM exhibits a lower increment. Steel slag RAM involving 50% RAP content reaches a spatter loss of 5.5% and only increases by 1.9% in comparison with virgin asphalt mixture. This indicates that on the one hand, aged asphalt leads to a reduction in the adhesion of the aggregate to the asphalt in RAM, causing the surface binder to fall off under the action of water. On the other hand, the viscosity of the aged asphalt is restored by the action of the rejuvenator, which leads to an improvement effect of bond performance for overall asphalt in RAM. Comparative analysis of spatter loss results verified that steel slag RAM demonstrates superior moisture susceptibility than basalt RAM. Furthermore, the rising RAP content will not cause structural damage to RAM with steel slag and basalt due to their low spatter loss.

### 3.3. High Temperature Stability Performance

**Figure 7:**
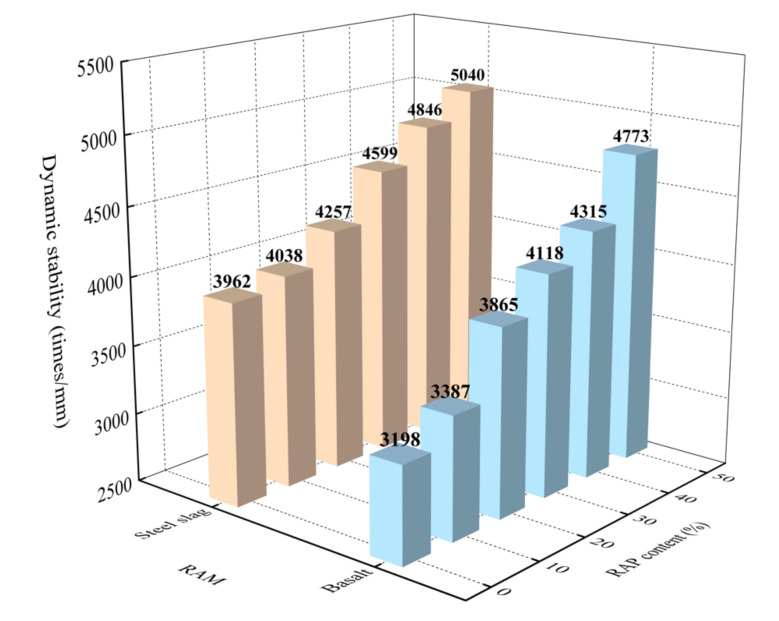
Dynamic stability results of RAM.

Figure 7 indicates the dynamic stability (DS) results of RAM. The participation of RAP boosts the DS of RAM and elevate its high temperature stability performance. It can be elaborated by the consequence that aged asphalt with high softening point and stiffness can prominently reduce the rutting depth of RAM and enhance its anti-rutting performance. Steel slag RAM reveals larger DS than RAM prepared with basalt, indicating its superior rutting resistance. This is attributed to the outstanding mechanical properties and abundant texture index of steel slag. The DS of steel slag RAM with 50% RAP is 5040 times/mm, which is 1.27 times that of the virgin asphalt mixtures. Steel slag and basalt RAM all exhibit DS much higher than 2400 times/mm (minimum index in standard) [38], representing their excellent high temperature performance.

### 3.4. Low Temperature Performance

The flexural tensile strain of asphalt mixture can effectively reflect the possibility of brittle fracture at low temperature and characterize its crack resistance. Asphalt mixture with greater flexural tensile strain means the superior low-temperature crack resistance. The crack resistance indexes from bending test results of RAM are provided in Table 3. As the RAP content rises, the maximum load, tensile strength and tensile strain of RAM ascend in contrast to stiffness modulus, resulting in reducing effect on low temperature performance. It can be explained by the fact that RAM with high RAP content displays lower plasticity and is incline to become hard and brittle, which weakens the resistance to low temperature deformation [42]. Comparison results of steel slag and basalt RAM state that involving steel slag can elevate the flexural tensile strength and tensile strain of RAM. Steel slag RAM with 50% RAP reveals a tensile strain of 2548.4 με. While the corresponding index is 2323.1 με for basalt RAM, which is less than the minimum value of 2500 με in the winter cold area in the specification. This indicates that when RAP content increases to 50%, steel slag RAM can be applied in severe cold areas instead of basalt RAM.

### 3.5. Skid Resistance

**Figure 8:**
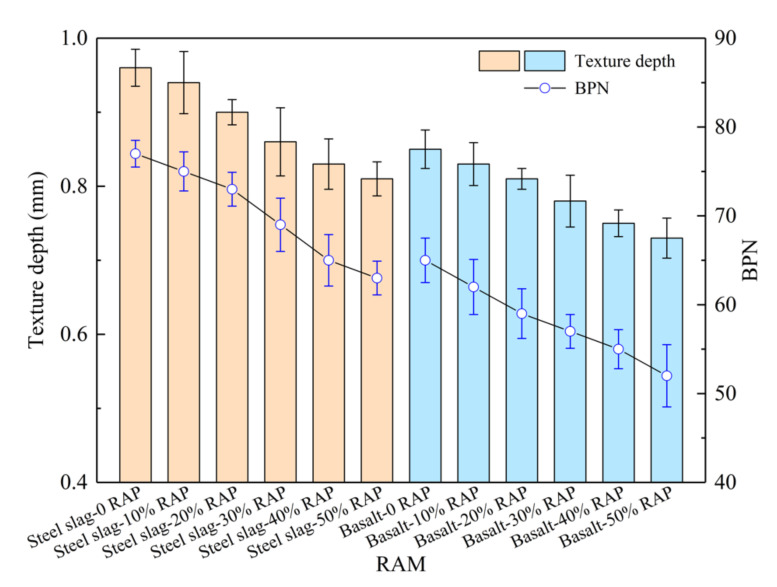
Texture depth and British Pendulum Number (BPN) results of RAM.

The texture depth and BPN results of RAM are illustrated in Figure 8. Virgin steel slag asphalt mixture demonstrates the highest texture depth and BPN, which reach to 0.95 mm and 77. Incorporating RAP decreases the texture depth and BPN of RAM. This is attributed to the deterioration of polished and weared stone value of RAP aggregate. When the same RAP dosage is involved, steel slag RAM displays larger texture depth and BPN than basalt RAM, indicating its superior skid resistance. This is attributed to the rich texture and impaction structure of steel slag [32]. Steel slag RAM with 50% RAP content exhibits the texture depth of 0.81 mm and BPN of 63, which far exceed the minimum value of 0.55 mm and 45 in requirements of the specification [38]. Texture depth of basalt RAM reduces from 0.86 mm, 0.83 mm, 0.81 mm, 0.78 mm and 0.75 mm to 0.73 mm as RAP content rises from 0 to 50% with an interval of 10%. BPN of basalt RAM with 50% RAP decreases by 20% and only reaches to 52, which is lower than 11 that of steel slag RAM and also arrival design constrain.

### 3.6. Durability

#### 3.6.1. Fatigue Resistance

**Figure 9:**
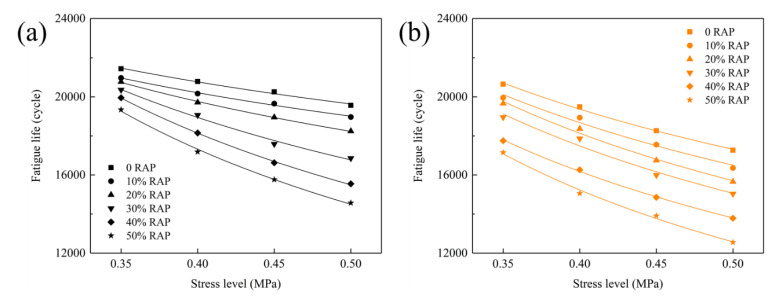
Fatigue life curve of RAM: (**a**) steel slag; (**b**) basalt.

Fatigue life, as the critical assessment indicator, can reflect the number of stress cycles of sample experienced failure. The fatigue life curves of RAM are depicted in Figure 9. Both of steel slag RAM and basalt RAM reveal a downward trend of fatigue life as RAP dosage increases. The conclusion is inconsistent with Yin et al.’s results [12]. This is due to the increase in the stiffness of blended asphalt and reduction in the response rate to stress for RAM [43]. Under the same RAP content, steel slag RAM embodies the higher fatigue life than basalt RAM, representing its superior fatigue performance.

Fitting coefficient of fatigue equation for RAM are presented in Table 4. The satisfactory correlation is found in all RAM samples for their high correlation coefficient. RAM with larger K and smaller n means preferable fatigue resistance. Steel slag virgin asphalt mixture possesses the highest K value, indicating its excellent fatigue performance. The addition of RAP decreases the K value and boosts n value of steel slag RAM. Compared with basalt RAM, incorporating steel slag in RAM can elevate its K value and fatigue resistance. This conclusion is match to the analysis results of fatigue life. This is because steel slag emerges better interfacial adhesion for its high angularity and texture.

#### 3.6.2. Hamburg Wheel Tracking Test

**Figure 10:**
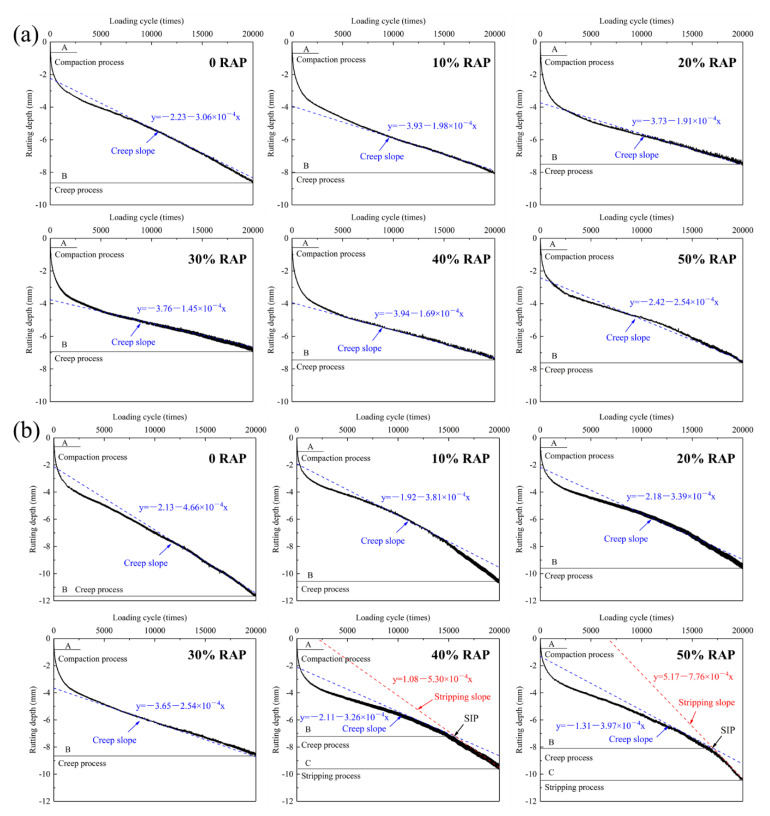
Deformation development of RAM with the increasing loading cycle: (**a**) steel slag; (**b**) basalt.

The Hamburg wheel tacking (HWT) test can comprehensively evaluate the rutting resistance, moisture susceptibility and stripping resistance under the coupling action of temperature and load. The deformation development of RAM with the ascending loading cycle are presented in Figure 10. The compaction process and creep process are discovered in steel slag RAM, while the stripping process only appears in basalt RAM with 40% and 50% RAP. The creep slope and stripping slop represent the deformation rate of RAM in rutting deformation and moisture damage, and larger values imply higher possibility of rutting destruction and moisture damage. The stripping inflection point (SIP), as a shift point from creep process to stripping process, its presence means the beginning of asphalt film peeling off from the surface of the aggregate [16]. As shown in Figure 10, all steel slag RAM do not reach the SIP. While SIP is observed in basalt RAM with 40% and 50% RAP, indicating their inferior durability.

Table 5 summarizes the HWT test results of RAM. Basalt virgin asphalt mixture reveals the largest rutting depth of 11.69 mm after 20,000 cycles loading. Under the same RAP dosage, steel slag RAM exhibits the lower rutting depth and creep slope than basalt RAM. When RAP content is lower than 40%, the rutting depth and creep slope of RAM reduce as the ascending RAP content, and then start to elevate after the RAP content reaches 40%. This is attributed to the greater attenuation function of RAP on water stability than its enhancement effect on rutting resistance for RAM with over 40% RAP content. Steel slag RAM with 50% RAP demonstrates the rutting depth of 7.60 mm and creep slope of 2.54 × 10^−4^, which are lower than corresponding values of 10.46 mm and creep slope of 3.97 × 10^−4^ for basalt RAM. It is worth mentioning that basalt RAM with 40% RAP content undergoes the stripping process under the cycles loading of 15,589 times and reaches the SIP. As the RAP dosage rises to 50%, the SIP moves back and appears at 17,065 cycles loading. The reason is that the ascending RAP content retards the cycles loading of SIP occurrences for RAM, but also elevates its stripping slop, resulting in the dramatic increase in the rutting depth at a later stage. Comparative analysis of HWT test results of RAM with steel slag and basalt state that involving steel slag endows the superior durability than basalt in high temperature and water environment.

### 3.7. Comparison Analysis Based on Radar Chart Evaluation Method

The radar chart evaluation method is conducted to quantitatively compare the effect of steel slag and basalt on the performance of RAM. The nine evaluation indicators, including residual Marshall stability, tensile strength ratio, spatter loss, dynamic stability, tensile strain, texture depth, British Pendulum Number, intercept K of fatigue equation, absolute value of creep slope in HWT test, were used in radar chart. They were denoted as RMS, TSR, SL, DS, TS, TD, BPN, Intercept K, CS, respectively. Table 6 provides the nine indicators of twelve groups in matrix A. Then the indicators are standardized and non-linear transformed referring to Equations (1) and (2), as shown in Table 7.

**Figure 11:**
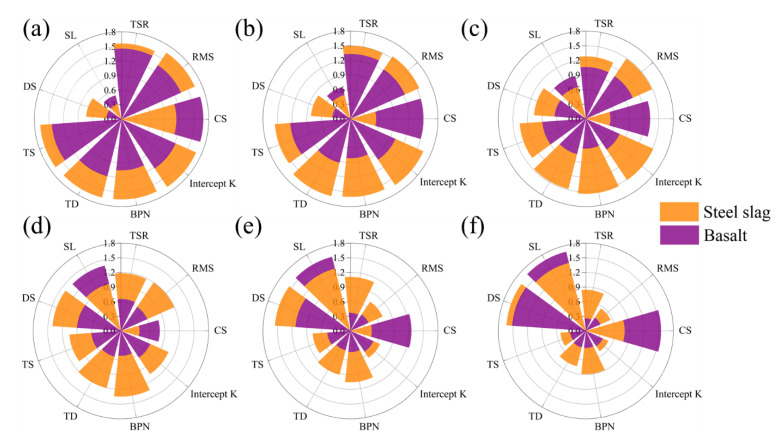
Radar charts for RAM with different RAP content: (**a**) 0 RAP; (**b**) 10% RAP; (**c**) 20% RAP; (**d**) 30% RAP; (**e**) 40% RAP; (**f**) 50% RAP.

Figure 11 summaries the radar charts for RAM with different RAP content. The discrepancies in the enhancement effect of steel slag are presented clearly. Incorporating steel slag reveals a significant improvement on RMS, TSR, DS, TS, TD, BPN and Intercept K in contrast to SL and CS. The appropriate RAP content in steel slag RAM can be identified from the charts considering the improvement of a certain performance indicator.

**Figure 12:**
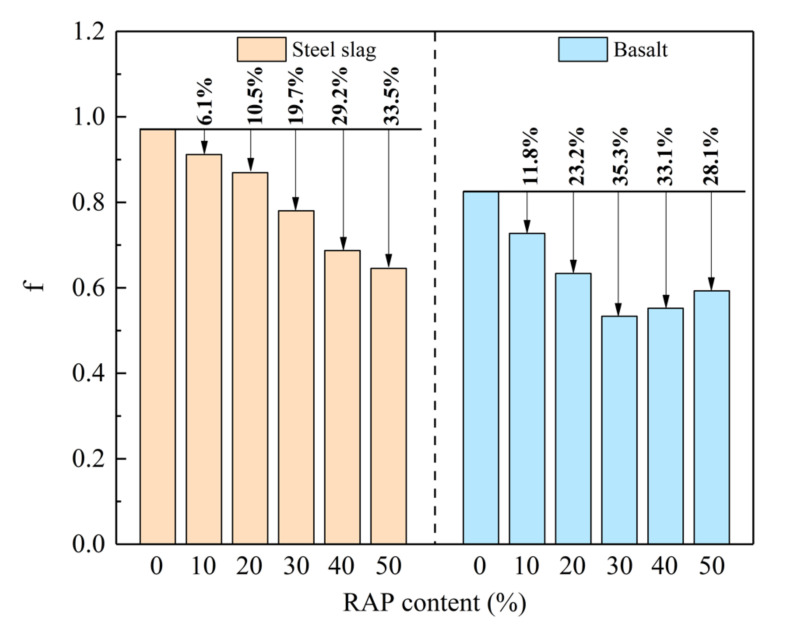
Comprehensive evaluation index of steel slag and basalt RAM.

According to Equations (3)–(7), the characteristic vectors (*u_i_*) and evaluation vectors (*ν_i_*) in matrices can be calculated, as listed in Table 8. Calculation results of comprehensive evaluation function (f) for twelve groups are elaborated in Figure 12. It is indicated that steel slag virgin asphalt mixture reveals the largest f value of 0.9710. The addition of RAP decreases f value of RAM with steel slag. When RAP dosage elevates from 0 to 50% with an interval of 10%, the f value of steel slag RAM are 0.9119, 0.8694, 0.7801, 0.6872 and 0.6456 with a decrement of 6.1%, 10.5%, 19.7%, 29.2% and 33.5%. Distinct from steel slag RAM, basalt RAM exhibits an upward and then downward trend as the increasing RAP dosage. This is due to the greater enhancement effect of RAP content on DS of basalt RAM than steel slag RAM. The f value of basalt RAM with 50% RAP content reduces by 28.1% and reaches to 0.5928. While RAM incorporated with steel slag possesses the larger f value than basalt under the same RAP content.

## 4. Conclusions

In this study, the pavement performances of recycled asphalt mixtures (RAM) with steel slag and basalt were examined using volume performance, moisture susceptibility, high temperature stability performance, low temperature performance, skid resistance and durability. Then, an improved radar chart evaluation method was applied to quantitatively assess their comprehensive performance. The following conclusions can be summarized.

- The incorporation of steel slag elevates the residual Marshall stability (RMS), tensile strength ratio (TSR) and diminishes the Cantabro spatter loss of RAM, endowing its superior moisture susceptibility than basalt RAM. Steel slag RAM involving 50% RAP dosage demonstrates a RMS of 90.5%, TSR of 89.3% and spatter loss of 5.5%, which remains at a high level;
- Steel slag RAM reveals larger dynamic stability and tensile strain in comparison to basalt RAM, representing its better rutting resistance and low temperature cracking resistance. The texture depth and BPN of steel slag RAM are 0.81 mm and 63, which are higher than those of basalt RAM by 0.08 mm and 11, respectively;
- RAM with steel slag embodies the larger K value and higher fatigue life, representing its superior fatigue performance compared with basalt RAM. All steel slag RAM do not reach the stripping process and stripping inflection point, which exhibit the lower rutting depth and creep slope than basalt RAM, resulting in preferable durability under high temperature and water condition;
- An improved radar chart evaluation method is capable of quantitatively assessing the discrepancies in the improvement effect of steel slag for RAM. Steel slag virgin asphalt mixture possesses the largest comprehensive evaluation function (f) of 0.9710, and the ascending RAP content diminishes the f value of steel slag RAM. Steel slag RAM displays larger f value compared with basalt RAM under the same RAP dosage, indicating its more desirable comprehensive road performance.

The above conclusions have verified that incorporating steel slag reinforces comprehensive road performance of recycled asphalt mixtures, which is conducive to promoting environmental sustainability. Further study is essential to investigate the engineering implementation and energy consumption of recycled asphalt mixtures involving steel slag.

## Figures and Tables

**Table 1:** The properties indexes of steel slag and basalt.

		Types	Steel Slag	Basalt
Indexes				
Apparent specific gravity	Particle sizes (mm)	9.5–16	3.369	2.983
		4.75–9.5	3.327	2.974
		2.36–4.75	3.138	2.972
Water absorption (%)	Particle sizes (mm)	9.5–16	1.59	0.58
		4.75–9.5	2.38	0.73
		2.36–4.75	2.82	1.04
Los Angeles abrasion (%)			13.9	20.7
Crushed value (%)			16.8	17.2
Polished value (%)			52	46
Adhesion level			5	4
Free-CaO content (%)			2.135	-

**Table 2:** Volume performance parameters of RAM.

Mixtures Styles	RAP Content (%)	Asphalt-Aggregate Ratio (%)	Bulk Density (g/cm^3^)	Air Voids (%)	VMA (%)	VFA (%)
Steel slag	0	4.9	2.653	4.2	14.1	70.3
	10	4.9	2.645	4.3	14.2	69.6
	20	4.9	2.637	4.5	14.1	68.2
	30	5.0	2.634	3.8	14.1	73.0
	40	5.0	2.624	4.0	14.2	72.0
	50	5.1	2.621	3.9	14.2	72.3
Basalt	0	4.7	2.520	3.9	14.0	72.2
	10	4.7	2.524	4.0	14.3	71.8
	20	4.8	2.528	3.8	14.0	72.6
	30	4.8	2.523	4.1	14.2	70.8
	40	4.8	2.527	4.0	14.3	72.3
	50	4.9	2.531	3.9	14.1	72.5

**Table 3:** The crack resistance indexes of RAM.

Mixtures Styles	RAP Content (%)	Maximum Load (N)	Tensile Strength (MPa)	Tensile Strain (με)	Stiffness Modulus (MPa)
Steel slag	0	1252.6	10.225	3269.2	3127.8
	10	1238.6	10.111	3102.2	3259.3
	20	1211.6	9.891	2955.8	3346.2
	30	1185.4	9.677	2813.5	3439.4
	40	1153.5	9.416	2695.9	3492.9
	50	1122.3	9.162	2548.4	3595.1
Basalt	0	1033.5	8.437	3006.7	2806.0
	10	1010.2	8.247	2892.2	2851.3
	20	975.3	7.962	2745.8	2899.6
	30	955.7	7.802	2603.5	2996.6
	40	906.5	7.400	2510.0	2948.2
	50	875.1	7.144	2323.1	3075.0

**Table 4:** Fitting coefficient of fatigue equation for RAM.

Mixtures Styles	RAP Content (%)	Fitting Formula (*N_f_* = K (*σ*)^−n^)				R^2^
		K		n		
		Value	Standard Deviation	Value	Standard Deviation	
Steel Slag	0	16,506.4	234.4	−0.251	0.016	0.9877
	10	15,716.9	198.5	−0.274	0.014	0.9919
	20	14,190.3	105.4	−0.362	0.008	0.9984
	30	11,486.9	344.0	−0.546	0.034	0.9888
	40	9495.0	111.5	−0.707	0.013	0.9990
	50	8346.9	204.7	−0.797	0.027	0.9965
Basalt	0	12,229.8	218.0	−0.502	0.020	0.9953
	10	11,233.9	500.1	−0.555	0.049	0.9764
	20	10,039.6	358.2	−0.646	0.039	0.9888
	30	9472.0	595.9	−0.669	0.070	0.9680
	40	8427.0	125.2	−0.712	0.017	0.9984
	50	6940.3	275.3	−0.858	0.044	0.9922

**Table 5:** The characterization parameters of RAM in HWT test.

Mixtures Styles	RAP Content (%)	Rutting Depth (mm)	Creep Slope (×10^−4^)	Stripping Slope (×10^−4^)	Stripping Inflection Point (SIP)
Steel slag	0	−8.61	−3.06	/	/
	10	−7.98	−1.98	/	/
	20	−7.51	−1.91	/	/
	30	−6.87	−1.45	/	/
	40	−7.40	−1.69	/	/
	50	−7.60	−2.54	/	/
Basalt	0	−11.69	−4.66	/	/
	10	−10.65	−3.81	/	/
	20	−9.58	−3.39	/	/
	30	−8.49	−2.54	/	/
	40	−9.15	−3.26	−5.30	X = 15,589, Y = −7.19
	50	−10.46	−3.97	−7.76	X = 17,065, Y = −8.08

**Table 6:** Evaluation indicators of RAM with different RAP content.

Mixtures Styles	RAP Content (%)	Water Stability			Rutting Resistance	Crack Resistance	Skid Resistance		Durability	
		RMS (%)	TSR (%)	SL (%)	DS (Times/mm)	TS (με)	TD (mm)	BPN	Intercept K	CS (×10^−4^)
Steel slag	0	95.9	95.2	3.6	3962	3269.2	0.95	77	16,506.4	3.06
	10	94.8	94.3	4.2	4038	3102.2	0.94	75	15,716.9	1.98
	20	94.4	92.2	4.5	4257	2955.8	0.90	73	14,190.3	1.91
	30	93.0	91.5	4.9	4599	2813.5	0.86	69	11,486.9	1.45
	40	91.3	91.0	5.3	4846	2695.9	0.83	65	9,495.0	1.69
	50	90.5	89.3	5.5	5040	2548.4	0.81	63	8,346.9	2.54
Basalt	0	93.6	93.8	4.2	3198	3006.7	0.86	65	12,229.8	4.66
	10	93.1	92.6	4.5	3387	2892.2	0.83	62	11,233.9	3.81
	20	92.5	90.7	4.8	3865	2745.8	0.81	59	10,039.6	3.39
	30	90.7	87.9	5.4	4118	2603.5	0.78	57	9472.0	2.54
	40	89.4	84.2	5.8	4315	2510.0	0.75	55	8427.0	3.26
	50	88.5	81.0	6.2	4773	2323.1	0.73	52	6940.3	3.97

**Table 7:** Evaluation indicators after standardization and non-linear transformation.

Mixtures Styles	RAP Content (%)	Water Stability			Rutting Resistance	Crack Resistance	Skid Resistance		Durability	
		RMS	TSR	SL	DS	TS	TD	BPN	Intercept K	CS
Steel slag	0	1.6550	1.5622	0.3213	0.7357	1.6839	1.6624	1.6544	1.6843	1.1337
	10	1.5449	1.4979	0.5064	0.8145	1.5587	1.6359	1.6043	1.6399	0.5300
	20	1.4894	1.2801	0.6726	1.0672	1.3631	1.4828	1.5400	1.5142	0.5055
	30	1.1971	1.1836	0.9927	1.4055	1.0601	1.2093	1.3490	1.0688	0.3821
	40	0.7223	1.1085	1.3161	1.5571	0.7816	0.9279	1.0554	0.6645	0.4393
	50	0.5567	0.8435	1.4367	1.6366	0.5252	0.7486	0.8900	0.5064	0.7985
Basalt	0	1.3427	1.4555	0.5064	0.3145	1.4437	1.2093	1.0554	1.2234	1.6884
	10	1.2234	1.3300	0.6726	0.3731	1.2406	0.9279	0.8113	1.0133	1.4978
	20	1.0563	1.0619	0.9053	0.6464	0.8956	0.7486	0.6121	0.7615	1.3232
	30	0.5929	0.6562	1.3803	0.9042	0.6060	0.5436	0.5131	0.6607	0.7985
	40	0.4071	0.3704	1.5663	1.1339	0.4781	0.4113	0.4367	0.5156	1.2538
	50	0.3288	0.2594	1.6752	1.5192	0.3244	0.3504	0.3531	0.3808	1.5470

**Table 8:** The characteristic vectors and evaluation vectors of matrices.

Mixtures Styles	RAP Content (%)	*u_i_* = [*A_i_*, *L_i_*]	*ν_i_* = [*ν_i_*_1_, *ν_i_*_2_]
Steel slag	0	[6.3817, 8.4425]	[1.0000, 0.9427]
	10	[5.6549, 7.9116]	[0.8861, 0.9385]
	20	[5.0353, 7.6201]	[0.7890, 0.9579]
	30	[4.0104, 6.8752]	[0.6284, 0.9685]
	40	[3.1861, 5.9849]	[0.4993, 0.9458]
	50	[2.8924, 5.5446]	[0.4532, 0.9197]
Basalt	0	[4.6372, 7.1484]	[0.7266, 0.9364]
	10	[3.5555, 6.3461]	[0.5571, 0.9494]
	20	[2.6350, 5.5926]	[0.4129, 0.9719]
	30	[1.9216, 4.6464]	[0.3011, 0.9455]
	40	[2.2578, 4.5889]	[0.3538, 0.8615]
	50	[2.8556, 4.7042]	[0.4475, 0.7853]

## Data Availability

Not applicable.
